# The Chloroplast Genome of *Elaeagnus macrophylla* and *trnH* Duplication Event in Elaeagnaceae

**DOI:** 10.1371/journal.pone.0138727

**Published:** 2015-09-22

**Authors:** Kyoung Su Choi, OGyeong Son, SeonJoo Park

**Affiliations:** Department of Life Sciences, Yeungnam University, Gyeongsan, Gyeongsangbuk-do, 712–749, Korea; University of Western Sydney, AUSTRALIA

## Abstract

Elaeagnaceae, which harbor nitrogen-fixing actinomycetes, is a plant family of the Rosales and sister to Rhamnaceae, Barbeyaceae and Dirachmaceae. The results of previous molecular studies have not strongly supported the families of Elaeagnaceae, Rhamnaceae, Barbeyaceae and Dirachmaceae. However, chloroplast genome studies provide valuable phylogenetic information; therefore, we determined the chloroplast genome of *Elaeaganus macrophylla* and compared it to that of Rosales such as IR junction and *infA* gene. The chloroplast genome of *Elaeagnus macrophylla* is 152,224 bp in length and the *infA* gene of *E*. *macrophylla* was psuedogenation. Phylogenetic analyses based on 79 genes in 30 species revealed that *Elaeagnus* was closely related to *Morus*. Comparison of the IR junction in six other rosids revealed that the *trnH* gene contained the LSC region, whereas *E*. *macrophylla* contained a *trnH* gene duplication in the IR region. Comparison of the LSC/IRb (J_LB_) and the IRa/LSC (J_LA_) regions of Elaeagnaceae (*Elaeagnus* and *Shephedia*) and Rhamnaceae (*Rhamnus*) showed that *trnH* gene duplication only occurred in the Elaeagnaceae. The complete chloroplast genome of *Elaeagnus macrophylla* provides unique characteristics in rosids. The *infA* gene has been lost or transferred to the nucleus in rosids, while *E*. *macrophylla* lost the *infA* gene. Evaluation of the chloroplast genome of *Elaeagnus* revealed *trnH* gene duplication for the first time in rosids. The availability of Elaeagnus cp genomes provides valuable information describing the relationship of Elaeagnaceae, Barbeyaceae and Dirachmaceae, IR junction that will be valuable to future systematics studies.

## Introduction

Rosales consists of approximately 7,700 species distributed into about 260 genera and nine families, Rosaceae, Ulmaceae, Cannabaceae, Moraceae, Urticaceae, Rhamaceae, Barbeyaceae, Dirachmaceae and Elaeagnaceae. Elaeagnaceae has been placed near Barbeyaceae, Dirachmaceae and Rhamnaceae [1]. Molecular analyses of Rosales has shown that relationships among Ulmaceae, Cannabaceae, Moraceae and Urticaceae were strongly supported [2]. However, phylogenetic relationships among Barbyaceae, Dirachmaceae, Rhamanaceae and Elaeagnaceae were weakly supported and not certain [2–4].

*Elaeagnus* L. belong to Elaeagnaceae, a small family that also contains *Hippophae* L and *Shepherdia* Nutt [5,6]. *Elaeagnus* consists of approximately 60 species distributed in Asia, Australia, southern Europe and North America [7]. *Elaeagnus macrophylla* is a popular ornamental plant valued for its aesthetic qualities and sweetly scented flowers. *E*. *macrophylla* is native to Eastern Asia.

The plant chloroplast (cp) genome consists of large inverted repeats (IRa, IRb) separated by a large single-copy (LSC) region and a small single-copy (SSC) region [8,9]. Approximately 100–120 genes are located in the cp genome, which is highly conserved [10]. However, some species in Asteraceae have been shown to have inversions [11], rearrangements have been observed in *Pelargonicum* [12], and gene loss and IR variations have been found in early-divergent eudicots [13, 14].

Recent studies of the IR region have enabled its use as an important marker describing relationships among plants. The IR region of most angiosperms ranges from 20 kb to 28 kb [12]. Previous analyses have shown various expansions or contractions of IR in some plants, such as 25 kb in *Cycas* [15], 114 bp in *Cryptomeria* [16] and 76 kb in *Pelargonium* [12]. Plunkett and Downie [17] reported IR expansion/contraction in Apioideae. IRa and IRb contain four junctions, J_LA_ (LSC/IRa border), J_SA_ (SSC/IRa border), J_SB_ (LSC/IRb border) and J_LB_ (SSC/IRb border). Most angiosperm plant IRb and IRa contain *rps19*- *rpl2* and *rps19*-*trnH*, respectively [18], while most monocot IRb and IRa contain *rps19*-*trnH* and *trnH*-*rps19*-*psbA*, respectively [19].

Previous studies have analyzed the complete chloroplast genome sequences of rosids and identified unique features such as inversion and gene transfer in their plastids [20]. Fagaceae of rosids showed changes in gene order in response to 51 kb inversions in *Glycine* and loss of the IR region in *Medicago* [20]. Genes of some rosid plastomes have been transferred to the nucleus [21,22]. For example, the *infA* gene (*Gossypium* [23], *Arabidopsis* [24], *Oenothera* [25] and *Lotus* [26]) and *rpl22* gene (*Castanea*, *Quercus* and *Passiflora* [22]) were transferred to the nucleus.

Here, we report the complete sequence of the chloroplast genome of *E*. *macrophylla* in Elaeagnaceae for the first time. In this review, we provide comparative analyses of the chloroplast genome of rosids species such as the *infA* gene, *rpl22* gene and IR junction. Specifically, we describe the structure of the chloroplast genome, IR junction characteristics and gene contents, which will better resolve phylogenetic relationships among rosids and Rosales.

## Materials and Methods

### Ethics, plant samples, sequencing, mapping and ananlysis

This research was approved by the Ministry of Environment in Korea (Daegu Regional Environmental Office). *Elaeagnus macrophylla* is not endangered or protected species. *Elaeagnus macrophylla* leaves were obtained from Dokdo Island (Korean Government), Korea. Total DNA was extracted using a DNeasy Plant Mini Kit (Qiagen Inc., Valencia, CA, USA) and quantified with a HiGenTM Gel & PCR Purification System (Biofact Inc., Daejeon, Korea). Genomic DNA was sequenced using an Ullumina Miseq sequencer (Illumina Inc., San Diego, CA). A total of 4,284,888 pair-end sequence reads of 300 bp were generated from the sequencing library with a median insert size of 500 bp, after which genome coverage was estimated using the CLC Genomics Workbench, v. 7.0.4 (CLC Bio, Aarhus, Denmark).

The complete chloroplast genome sequence was annotated using a Dual Organellar Genome Annotator [DOGMA] [27]. All of the identified tRNA genes were further verified using the corresponding structures predicted by tRNAscan-SE [28]. A circle cp genome map was drawn using the OGDRAW program [29]. Geneiou s v.6.1.7 [30] was employed to compare the cp genome of *E*. *macrophylla*, *Morus indica* and *Prunus kansuensis*.

### Phylogenetic analyses

A total of 79 genes sequences from 30 species (S1 Table) were aligned using MAFFT [31]. Phylogenetic analysis was conducted based on the maximum likelihood (ML) using the GTR+R+I model in RAxML v. 7.2.6 [32] and 1,000 bootstrap replicates. ML analysis resulted in a single tree with–lnL = 485,367.343.

### PCR amplification and comparative analysis of IR junctions

Six species of Elaeagnaceae (*Elaeagnus* and *Shephedia*) and six species of Rhamnaceae (*Rhamnus*) were evaluated (S2 Table) using the following primers specific for the LSC/IRb junction and IRa/LSC junction designed with Primer3 [33]: 1) *rps19*-*rpl2*: Forward, CGCTCGGGACCAAGTTACTA; Reverse, GGGTTATCCTGCACTTGGAA 2) *rpl2*-*psbA*: Forward, ATGTTGGGGTGAACCAGAAA; Reverse, GCTGCTTGGCCTGTAGTAGG. Total DNA was extracted as described by Allan et al. [34] and then subjected to PCR amplification. PCR cocktail (25μl) consisted of 250ng genomic DNA, 1X Diastar^TM^ *Taq* DNA butter, 0.2 mM of each dNTP, 10 pM of each primer and 0.025 U of Diastar^TM^ *Taq* DNA polymerase (SolGent Co., Korea). The amplification conditions were as follows: initial denaturation at 95°C for 2 min, followed by 35 cycle of 95°C for 20sec, 56°C for 40sec, and 72°C for 50sec, with a final extension at 72°C for 5min, after which samples were held at 8°C. Amplification products were purified using a HiGenTM Gel & PCR Purification System (Biofact Inc., Daejeon, Korea). Nucleotide sequences of the *rps19*-*rpl2* region and *rps19*-*psbA* regions were aligned with Geneious v. 6.1.7 [30].

## Results

### Comparison of the chloroplast genome of *Elaeagnus macrophylla* to those of other rosids

**Fig 1:**
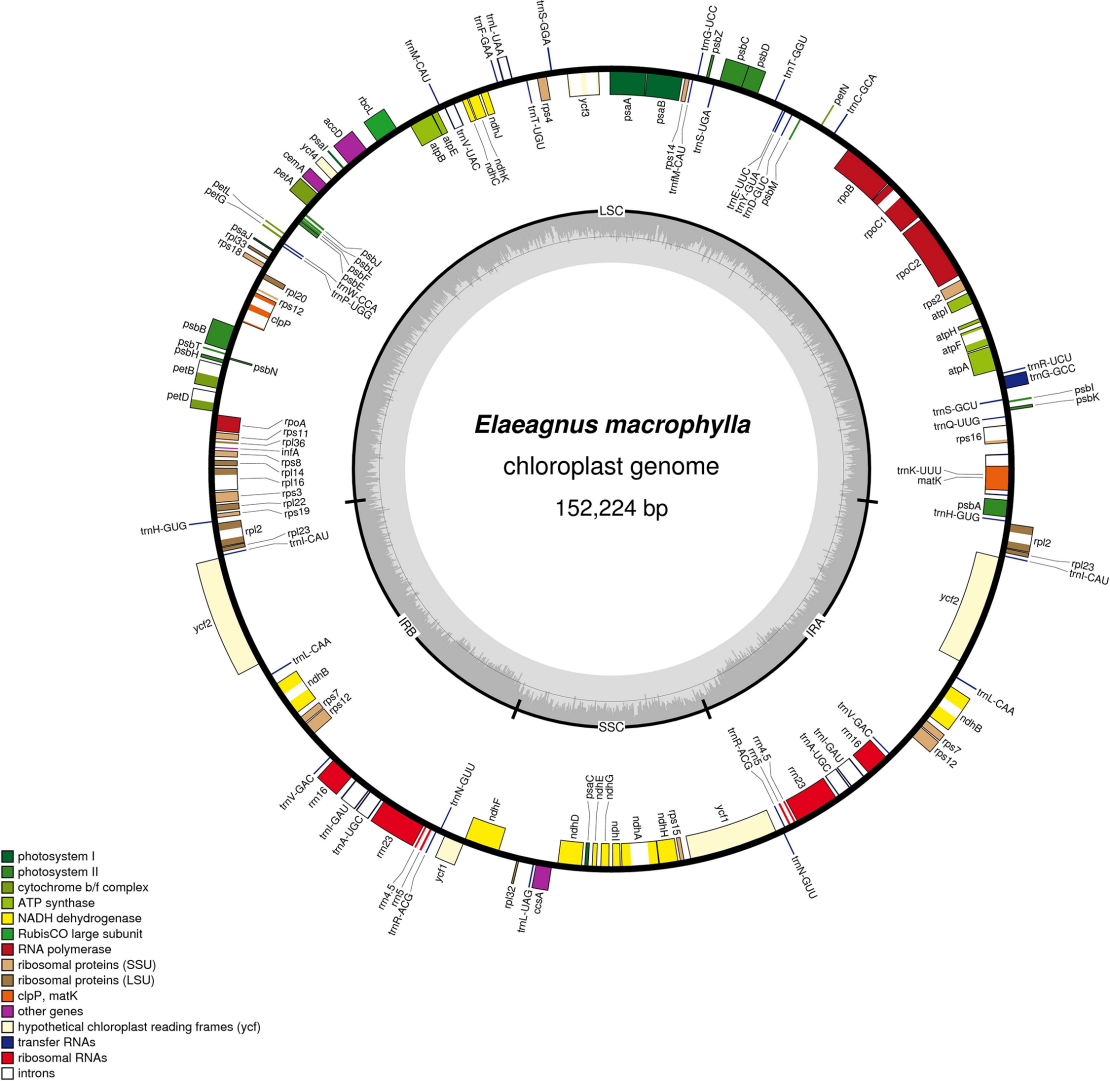
Complete chloroplast genome of *Elaeagnus macrophylla*. Genes drawn inside the circle are transcribed clockwise, while outside genes are counterclockwise. The gray plot in the inner circle corresponds to the GC content.

The cp genome sequence of *E*. *macrophylla* was submitted to GenBank and assigned accession number KP211788. The cp genome contains 152,224 bp, the LSC has 82,136 bp, the SSC has 18,278 bp and the IR has 25,905 bp (Fig 1). We identified 113 unique genes in *E*. *macrophylla*, 80 protein coding genes, 29 transfer RNA (tRNA) genes and 4 ribosomal RNA (rRNA) genes. The genome consisted of 73.4% coding genes (111,792 bp), including 60.5% protein-coding genes (92,119 bp), 7% tRNA genes (10,625 bp) and 5.9% rRNA genes (9,048 bp). Additionally, 18 genes encoded introns among unique genes of *E*. *macrophylla*, among which 12 are protein-coding genes and six are tRNA genes. Three protein coding genes include two introns (*clpP*, *ycf3* and *rps12*), and the overall A+T content of *E*. *macrophylla* is 63.9%. The A+T percentages are higher in the SSC region (69.4%) than the LSC (65%) and IR regions (57.3%).

Previous studies, *E*. *macrophylla* belong to Rosales [2] and complete chloroplast genome of Rosales studied in *Morus indica* (NC_008359) and *Prunus kansuensis* [22]. Therefore, the genome features of *E*. *macrophylla* were compared to *M*. *indica* and *P*. *kansuensis* (Table 1). The total size of the chloroplast genome was longer in *P*. *kansuensis* (157,790 bp) than *E*. *macrophylla* (152,224 bp) and *M*. *indica* (158,484 bp). The length of the LSC region (82,136 bp to 87,386 bp) differed significantly from the SSC (18,278bp to 19,745 bp) and IR regions (26,387 bp to 26,678 bp). The average AT content of the Rosales cp genome is 63%, with the highest being observed in *M*. *indica* (63.63%).

**Table 1:** General features of *Eleaegnus macrophylla* and comparison to those of Rosales.

Genome features	*E*. *macrophylla*	*Morus indica*	*Prunus kansuensis*
Total length (bp)	152,224 bp	158,484 bp	157,790 bp
LSC length	82,136 bp	87,386 bp	85,811 bp
SSC length	18,278 bp	19,742 bp	19,151 bp
IR length	25,905 bp	25,678 bp	26,387 bp
A+T content	63.9%	63.63%	63.2%
Number of genes	129	129	129
Number of gene duplicated in IR^a^	17	16	16

*Prunus kansuensis* has re-annotation in this study.

^a^ *rps12* is not included in this number; only genes completely duplicated are included.

### Genes of *infA* and *rpl22* in rosids

**Fig 2:**
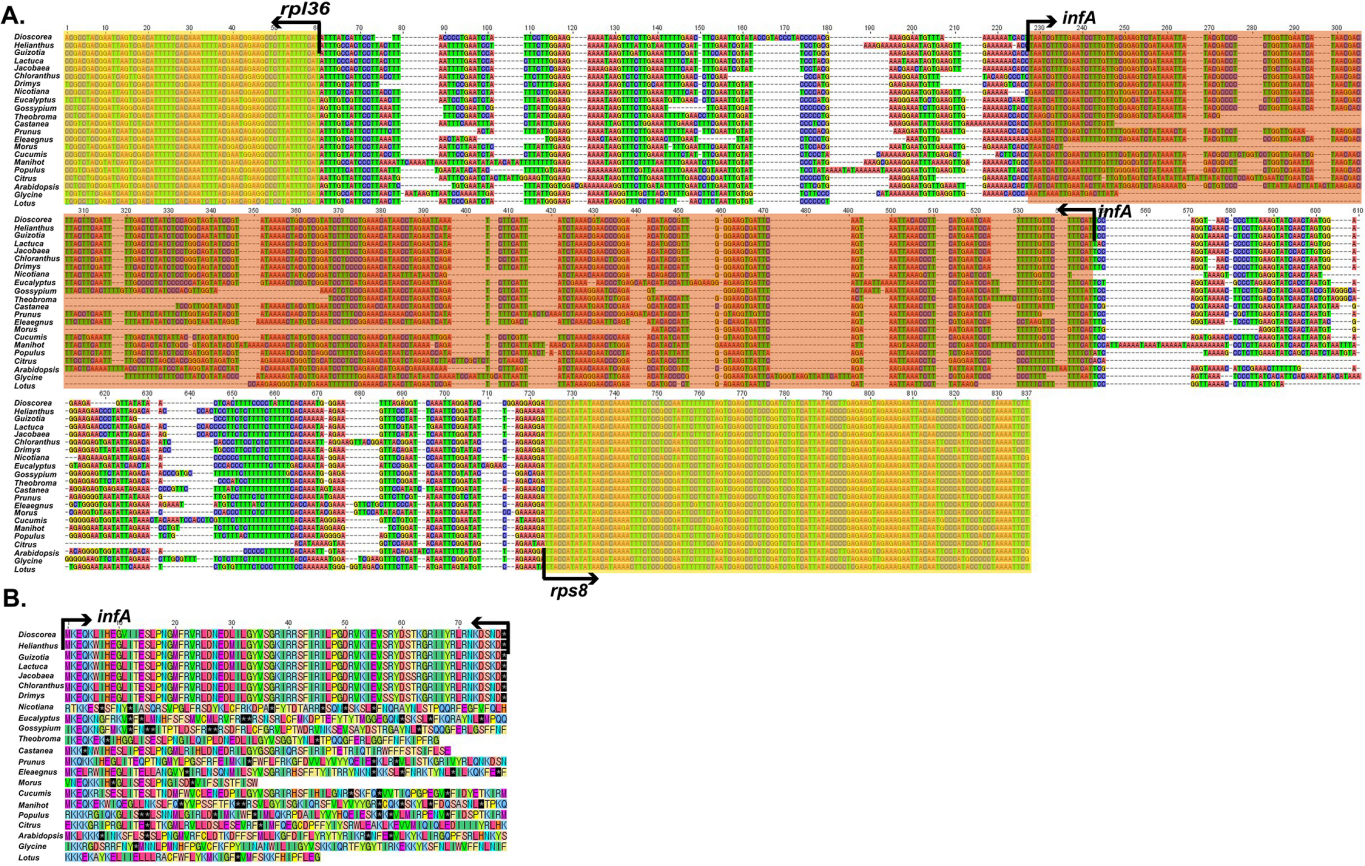
*rpl36*-*rps8* region sequence alignment of 22 species in angiosperms. A. The *rpl36*-*rps8* region aligned nucleotide sequence. B. Amino acid sequence alignment of *infA* (red box in Fig 2A). *Dioscorea* in Monocots; *Helianthus*, *Guizotia*, *Lactuca*, *Jacbaea*, and *Nicotiana* in Astrids; *Chloranthus* in Chloranthales; *Drymis* in Magnoliids; *Eucalyptus*, *Gossypum*, *Theobroma*, *Castanea*, *Prunus*, *Morus*, *Cucumis*, *Manihot*, *Populus*, *Citrus*, *Arabidopsis*, *Glycine*, and *Lotus* in Rosids.

The functional gene sequences of *infA* and *rpl2*2 are highly variable in rosids. The *infA* gene of rosids differs from that of most asterids (*Helianthus*, *Guizotia*, *Lactuca* and *Jacobaea*), monocots (*Dioscorea*), magnoliids (*Drimys*) and chloranthales (*Chloranthus*) owing to the presence of the pseudogene, *infA*, in *Elaeagnus* and other rosids. However, other plants such as *Dioscorea*, *Helianthus*, *Guizotia*, *Lactuca*, *Jacobaea*, *Chloranthus* and *Drimys* encode homologous sequences (Fig 2).

The *rpl22* gene of *Arabidopsis*, *Glycine* and *Lotus* showed an internal stop codon. However, the *rpl22* gene of the *rpl22* gene of other plants consists of the start codon (methionine) to stop codon (data not shown). Nevertheless, the size of the *rpl22* gene in another 18 species ranged from 252 bp in *Cucumis* to 552 bp in *Guizotia*, while it was 423 bp in *Elaeagnus*.

### Comparison of IR region in Rosids

**Fig 3:**
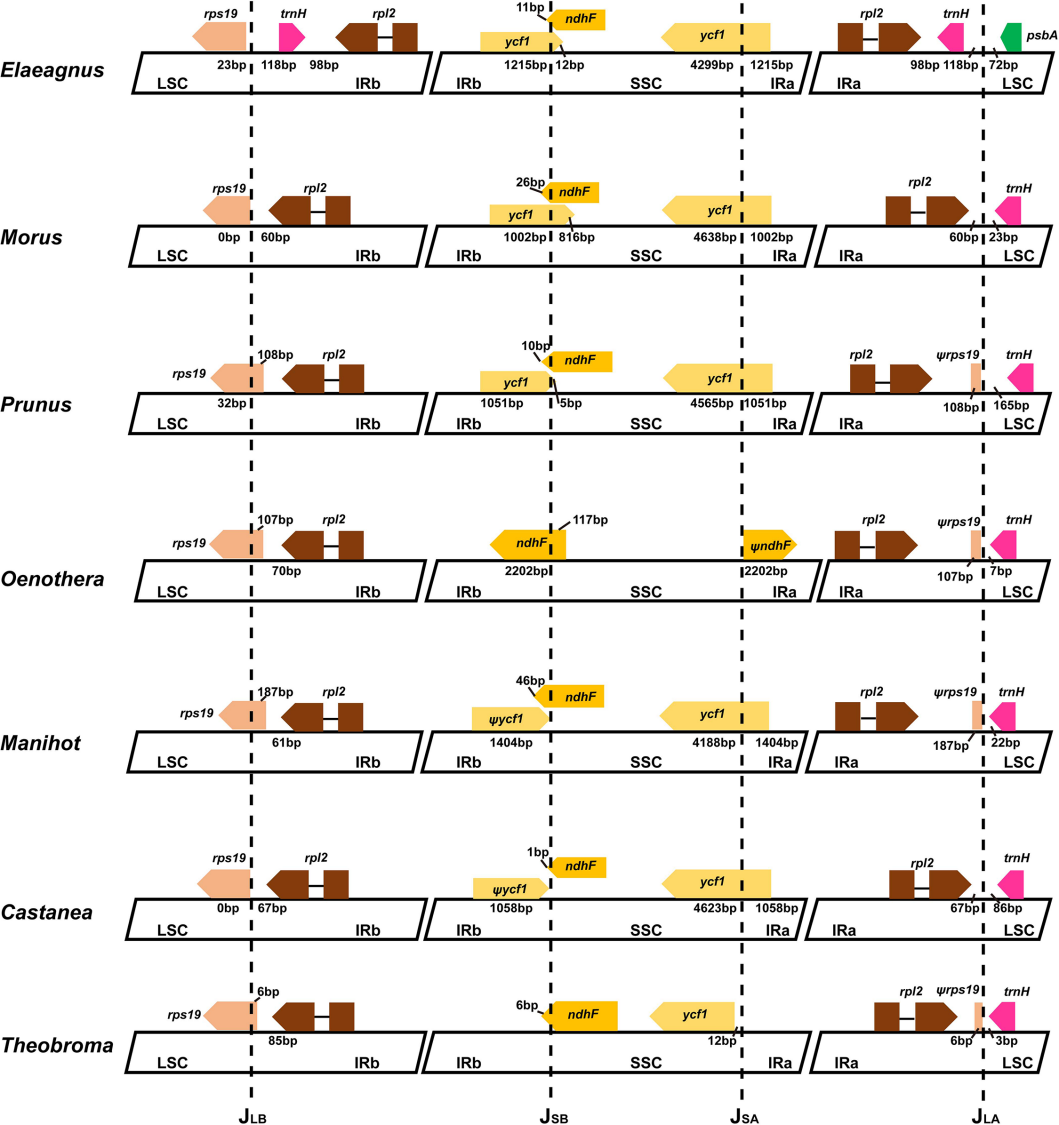
Comparison of four junctions (LSC/IRb, IRb/SSC, SSC/IRa and IRa/LSC) among eight rosid genomes.

We compared the IR region of seven species (*Elaeagnus*, *Morus*, *Prunus*, *Oenothera*, *Manihot*, *Castanea* and *Theobroma*) of rosids (Fig 3). In *Prunus*, *Oenothera*, *Manihot* and *Theobroma*, J_LB_ occur within the *rps19* gene, resulting in partial duplication of this gene in IRa at J_LA_ (108 bp, 107 bp, 187 bp and 96 bp, respectively). Nevertheless, *rps19* is not duplicated in *Morus* and *Castanea*. In contrast, differing gene arrangement such as complete duplication of *trnH* was observed in the LSC/IRb and LSC/IRa border regions of *Elaeagnus*.

The *ycf1* gene is duplicated in the IRb/SSC (J_SB_) and SSC/IRa (J_SA_) borders of rosids. This gene duplication varies from 1,002 bp (*Morus*) to 1,404 bp (*Manihot*). In *Oenothera*, 2022 bp of *ndhF* were duplicated in the IR region. Conversely, the *ndhF* and *ycf1* genes of *Theobroma* are not duplicated in the IR.

### Phylogenic analyses of *Elaeagnus* and Rosids

**Fig 4:**
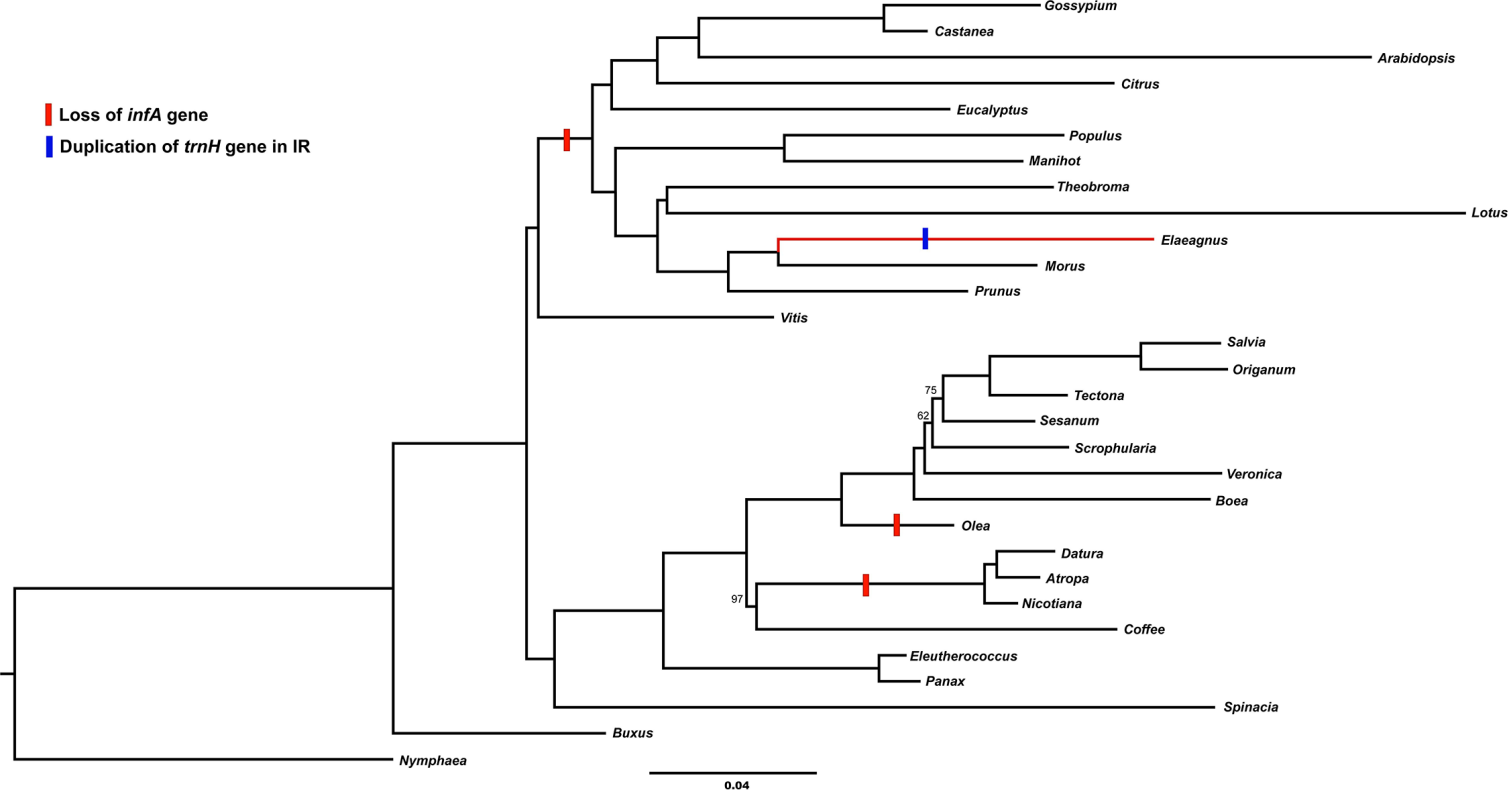
Maximum likelihood tree based on 79 coding genes. The maximum likelihood phylogram has an ML value of–lnL = 48,5367.343. The red box indicated loss of the *infA* gene and the blue box indicated duplication of the *trnH* in the IR region.

Phylogenic analysis was conducted using a gene data matrix based on 79 genes from 30 species with 75,370 bp aligned nucleotides (Fig 4). Rosids and asterids form two well supported monophyletic sister groups with strong support (100% bootstrap values). Rosids are a well-defined group with two strongly supported clades: Fabidae (*Prunus*, *Morus*, *Elaeagnus*, *Lotus*, *Theobroma*, *Manihot* and *Populus*); and Malvidae (*Gossypium*, *Castanea*, *Arabidopsis*, *Citrus* and *Eucalyptus*). The results of the present study confirmed that the genus *Elaeagnus* belongs to Fabidae and forms a sister relationship with *Morus* with 100% bootstrap values.

The *infA* gene has been lost from many angiosperms in land plants, and Millen et al. [21] suggested functional replacement of a nucleus copy. Our results indicate that the *infA* gene has been lost from rosids (including *Elaeagnus*). We also found that *trnH* duplication of the IR region was only present in *Elaeagnus*.

### Comparison of the *trnH* gene in the IR region between Rhamnaceae and Elaeagnaceae

A previous study reported that Elaeagnaceae is closely related to Rhamnaceae, Dirachmaceae and Barbeyaceae [2,14,35]. In the present study, the chloroplast genome data revealed that the gene order in the LSC/IRb region of *E*. *macrophylla* continued to *rps19*, *trnH* and *rpl2*, while the IRa/LSC region continued to *rpl2*, *trnH* and *psbA*. Therefore, we compared the LSC/IRb (J_LB_) and the IRa/LSC (J_LA_) regions of Elaeagnaceae (*Elaeagnus* and *Shephedia*) and Rhamnaceae (*Rhamnus*). Fourteen species of Elaeagnaceae (*Elaeagnus* and *Shephedia*) and Rhamnaceae (*Rhamnus*) does experiments and aligned the sequences of *rps19*-*rpl2* (J_LB_ region) and *rpl2*-*psbA* (J_LA_) regions.

**Fig 5:**
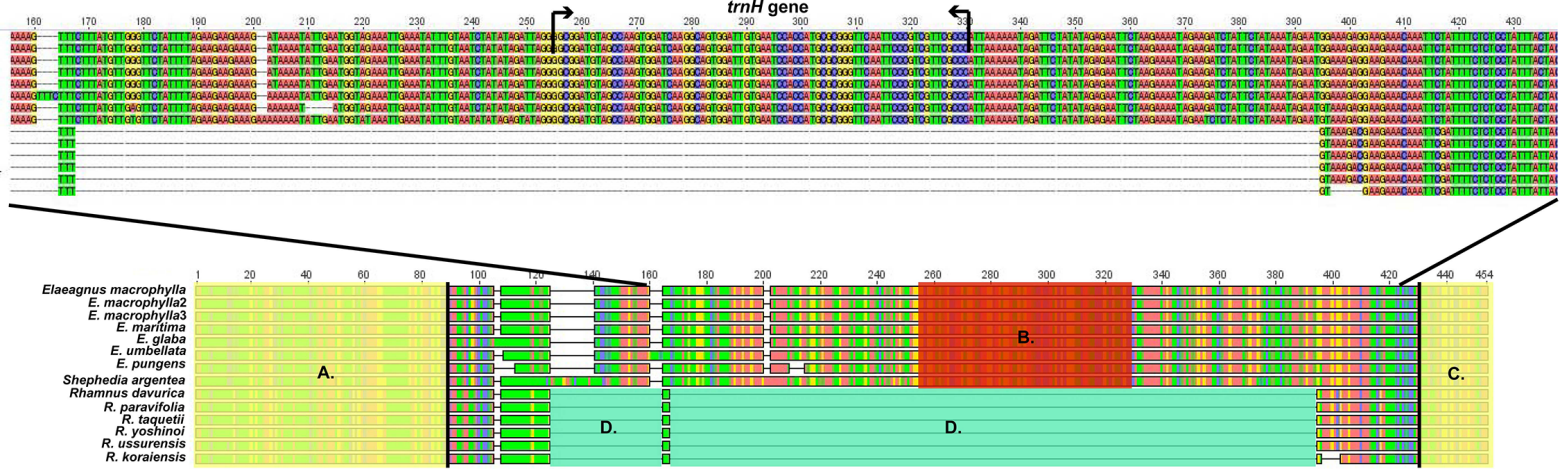
Comparison of the *rps19*-*rpl2* region sequences between Elaeagnaceae (*Elaeagnus* and *Shephedia*) and Rhamnaceae (*Rhamnus*). A: partial *rps19* gene B: *trnH* gene C: partial *rpl2* gene D: gaps.

**Fig 6:**
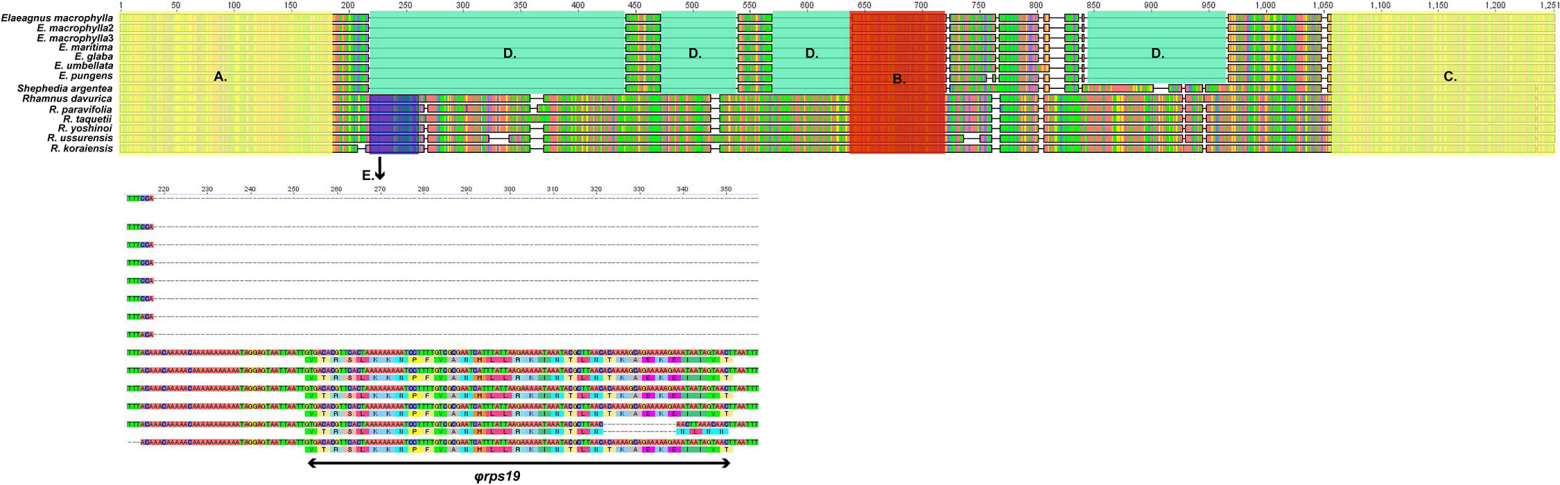
Comparison of *rpl2*-*psbA*region sequences between Elaeagnaceae (*Elaeagnus* and *Shephedia*) and Rhamnaceae (*Rhamnus*). A: partial *rpl2* gene, B: *trnH* gene, C: partial *psbA* gene, D: gaps, E: *ψrps19* gene. Below Fig indicates the nucleotide sequence and translation of *ψrps19* (annotation by DOGMA).

The *rps19*- *rpl2* region of Elaeagnaceae differed from that of Rhamnaceae (Fig 5). The *rps19* (Fig 5A) and *rpl2* (Fig 5C) regions of Elaeagnaceae were highly similar to those of Rhamnaceae, whereas the areas surrounding the *trnH* and *trnH* gene differed greatly between these families (Fig 5B and 5D). Additionally, the *rpl2*-*psbA* region (Fig 6) between Elaeagnaceae and Rhamnaceae could be distinguished by the *ψrps19* gene (Fig 6E). The *rpl2*, *trnH*, and *psbA* genes are conserved in Elaeagnaceae and Rhamaceae, whereas Elaeagnaceae has long gaps among coding genes and the *trnH* gene (*rpl2*-*trnH* and *trnH*-*psbA*).

## Discussion

Rosids comprise the largest clade among eudicots (20 orders and 140 families), and include plants that form nitrogen-fixing symbioses (Elaeagnaceae, Rhamnaceae, Rosaceae, and Ulmaceae) [36], as well as many important crops (Fabaceae (legumne) and Rosaceae (fruit crops)). Accordingly, rosids plants have been very well studied, and almost the entire chloroplast genome is known [14, 20, 36–39].

### Rosid gene contents

Previous studies revealed that the *infA* and *rpl22* genes and *atpF* intron have been lost or subjected to psuedogenation in rosids. Millen et al. [21] and Jansen et al. [22] found that the chloroplast genes, *infA* and *rpl22*, are transferred to the nucleus in rosids. However, the intron was lost from the *atpF* gene of Cassava (*Manihot esculenta*) [38]. Moreover, the *infA* gene has been independently lost multiple times from angiosperms and most rosids [22, 26]. Phylogenic studies placed *Elaeagnus* sister to *Morus* in the Rosales clade [2, 26], and complete chloroplast genome analysis of *Morus* did not reveal an *infA* gene [40]. The *rpl22* gene has been lost from Fabaceae (*Glycine* and *Medicago*) and Fagaceae (*Castanea* and *Quercus*), and these plants have been independently transferred to the nucleus [22].

Our results also revealed the putative loss or formation of a pseudogene of the *infA* gene in *E*. *macrophylla* (Fig 2B). Moreover, the loss of the *infA* gene of 12 rosids was observed in this study (Fig 4). Su et al. [41] showed that *Quercus*, *Francoa* and *Cucumis* contain intact *infA* genes; however, no *infA* gene was observed in *Cucumis* in the present study (Fig 4).

The *rpl22* gene of *E*. *macrophylla* is intact, while it was lost from *Arabidopsis*, *Glycine*, *Lotus* and *Castanea*, and present in varying lengths in 18 other species.

### Special event in the IR region of *Elaeagnus*

The chloroplast genome of land plants is highly conserved structurally, and the junction of large inverted repeats (IRs) is not essential to chloroplast genome function [18]. Because of black pine, *Conopholis* and *Phelipanche* of Orobanchaceae and *Erodium* was not present the IR region [42–44]. However, the IR region is a variable site on the chloroplast genome with useful features [17,45,46].

The gene arrangement of the IR region in most eudicots is different from that of monocots. The gene arrangement of basal plants and monocots in J_LB_ (LSC/IRb region) are *rpl23*, *rpl2*, *trnH*, *rps19* and *rpl22*, while gene arrangement in J_LA_ (IRa/LSC region) is *rpl23*, *rpl2*, *trnH*, *rps19*, and *psbA* [22, 41]. Thus, the *trnH* gene contains two IR regions. However, most eudicots do not undergo *trnH* gene duplication in the IR region. Nevertheless, the IR region border of most eudicots, including rosids plants, contains the *rps19* or *ψrps19* gene [14,20,22–25,38].

As shown in Fig 3, the LSC-IRb junction of the *Elaeagnus* species shows insertion of the *trnH* gene, whereas the other rosids species do not contain the *trnH* gene. Comparison of the LSC-IRb region of closely related species of Rhmanaceae revealed an approximately 600 bp gap after the *rps19* gene (Fig 5). In contrast, the IRa-LSC region contains 600bp gaps in Elaeagnaceae species. The *trnH* gene of Elaeagnaceae and Rhamnaceae is the same length, but Elaeagnaceae does not include the *rps19* gene (Fig 6). Consequently, Elaeagnaceae and Rhamancaea have different gene contents and arrangements in the IR region.

Comparisons of J_LB_ and J_LA_ in Rosales revealed that the *rps19* gene is not duplicated in *Morus*, whereas, *Prunus* contains a 108 bp duplication of the *rps19* gene. The gene *ycf1* is duplicated from 1,002 bp in *Morus* to 1,051 bp in *Prunus*. However, the *trnH* gene is duplicated in the J_LB_ (*rps19* is not duplicated) and J_LA_ border, and 1,215 bp of *ycf1* is duplicated in *Elaeagnus*. Hence, the IR length of *Elaeagnus* was longest in *Morus* and *Prunus*.

Wang et al. [19] has suggested two possible mechanisms of the evolution of IR expansions in Monocots. Wang et al. [19], double-strand break (DSB) events occurred within the IRb, after which the free 3’ end of the broken strand was repaired against the homologous sequence in IRa. The repaired sequence then extends over the original IR-LSC junction, reaching the area downstream of *trnH*, resulting in duplication of the *trnH* gene in the newly repaired IRb. Similarly, the IR region extends in Elaeagnaceae.

### Duplication of the *trnH* gene in Elaeagnaceae

**Fig 7:**
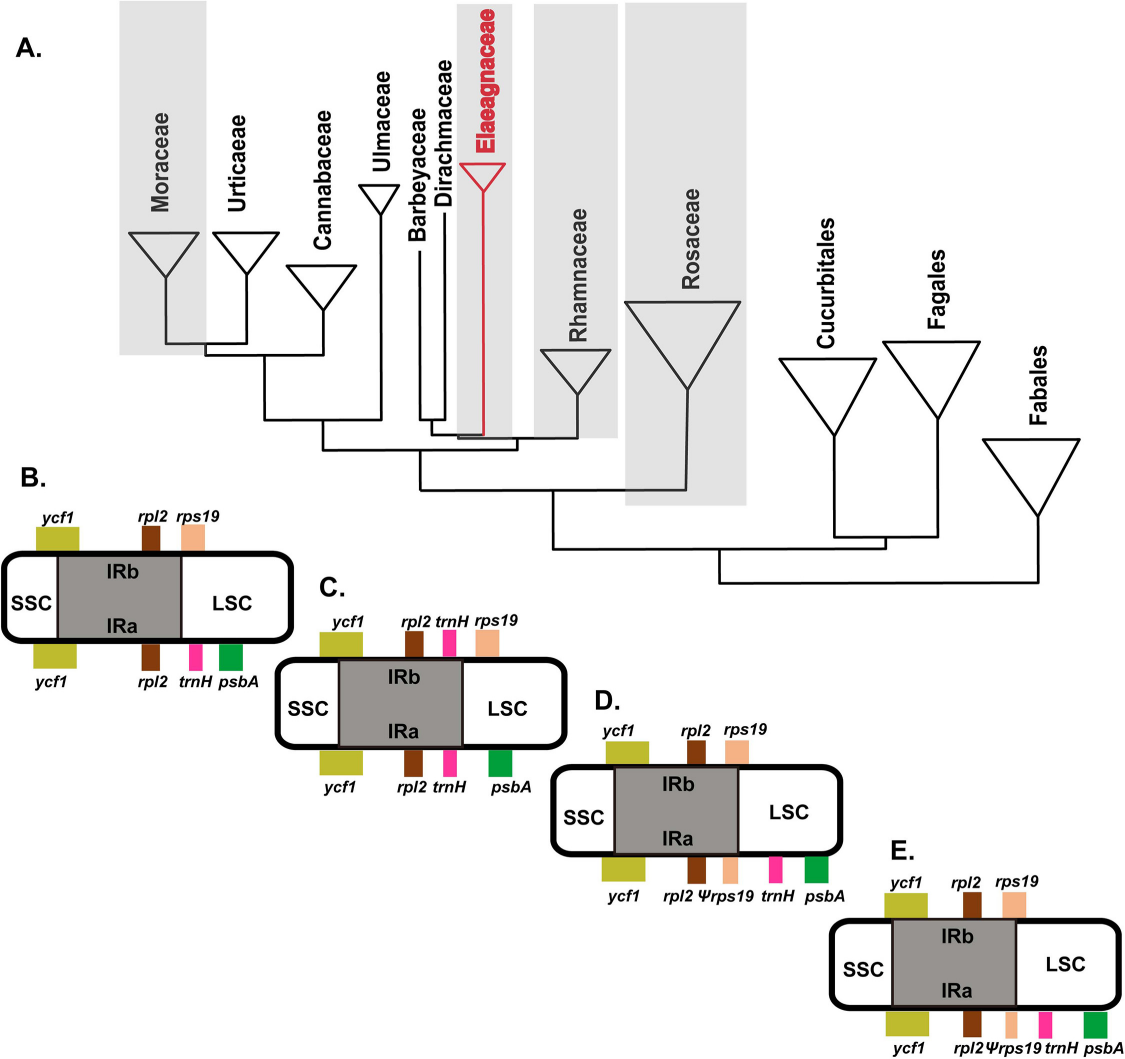
Duplication of *trnH* gene in Elaeagnaceae. A: Previous phylogenetic tree of Rosales (Zhang et al, [2]), B: Four junctions (LSC/IRb, IRb/SSC, SSC/IRa, and IRa/LSC) of *Mous* in Moraceae, C: Four junctions of *Elaeagnus* in Elaeagnaceae, D: Four junctions of *Rhamnus* in Rhamnaceae, E: Four junctions of *Prunus* in Rosaceae.

Our data analyses confirmed IR evolution in Rosales (Fig 7A). The incomplete *rps19* gene of *Prunus* in Rosaceae (Fig 7E) and *Rhamnus* in Rhamnaceae (Fig 7D) was duplicated in the IR region. Conversely, *Morus* in Moraceae did not contain a duplicated *rps19* gene in the IR region (Fig 7B). Only the Elaeagnaceae was duplicated in the *trnH* gene (Fig 7C). The *trnH* gene duplication is a useful marker in Rosales, such as Dirachmacae, Barbeyaceae and Elaeagnaceae. In a previous study, Richardson et al. [3] suggested a sister relationship between Rhamnaceae, Dirachmaceae and Barbeyaceae. In contrast, Zhang et al. [2] suggested a sister relationship among Elaeagnaeae, Dirachmaceae and Barbeyaceae, but this was not well supported in the Elaeagnaceae clade. Consequently, analyses of *trnH* duplication in the LSC/IRb junction and the IRa/LSC junction from different Moraceae and Elaeagnaceae would be of great value in systematics studies.

## Conclusions

Here, we present the complete chloroplast genome of *Elaeagnus macrophylla* and compare it to that of rosids. The *infA* gene has been lost from the chloroplast genome or transferred to the nucleus in angiosperms [21]. Most rosids, including *E*. *macrophylla*, show loss of the *infA* gene. The chloroplast genome consists of a LSC (Large Single Copy), SSC (Small Single Copy) and two IR (Inverted Repeat) regions. The IR region is between 20 and 30 kb in length in angiosperms, and clearly differs among closely related species. The IR region of *E*. *macrophylla* differs owing to *trnH* gene duplication. Phylogenetic analysis strongly supports a monophyletic group of Rosales (*Elaeagnus*, *Morus* and *Prunus*). Previous studies did not clearly support Eleaganaceae, Rhamnaceae, Dirachmaceae and Barbeyaceae in the molecular phylogenetic tree. In the present study, comparison of *trnH* gene duplication in two closely related families, Elaeagnaceae and Rhmanaceae, showed that no duplication occurred in Rhmanaceae, but that it occurred in Elaeagnaceae. Consequently, *trnH* gene duplication in Elaeagnaceae offers information that will be useful for systematics and elucidation of the relationship between Elaeagnaceae, Dirachmaceae and Barbeyaceae.

## Supporting Information

S1 Table Phylogenetic study taxa and Genebank accession numbers of references. (DOCX)

S2 Table IR junction analysis taxa and accession numbers. (DOCX)

## References

[1] Angiosperm Phylogeny Group (APG). An update of the Angiosperm Phylogeny Group classification for the orders and families of flowering plants: APGIII. Bot J Linn Soc. 2009; 161: 105–121.

[2] Zhang S-D, Soltis DE, Yang Y, Li D-Z, Yi T-S. Multi-gene analysis provides a well-supported phylogeny of Rosales. Mol phylogenet Evol. 2011; 60: 21–28. 10.1016/j.ympev.2011.04.008 21540119

[3] Richardson JE, Fay MF, Cronk QCB, Bowman D, Chase MA. A phylogenetic analysis of Rhamnaceae using *rbcL* and *trnL*-*F* plastid DNA sequences. Am J Bot. 2000; 87(9): 1309–1324. 10991902

[4] Soltis DE, Soltis PS, Chase MW, Markt ME, Albach DC, Zanis M et al Angiopserm phlogeny inferred from 18S rDNA, *rbcL*, and *atpB* sequence. Bot J Linn Soc. 2000; 133: 381–461.

[5] Mabberly DJ. The plant-book Cambridge University Press; 1998.

[6] Sun M, Lin Q. A revision of *Elaeagnus* L. (Elaeagnaceae) in mainland China. J Syst Evol. 2010; 48(5): 356–390.

[7] Heywood VH, Brummitt RK, Culham A, Seberg O. Flowering plant families of the world Kwe Publishing London UK; 2007pp. 135–136.

[8] Palmer JD. Contrasting modes and tempos of genome evolution in land plant organelles. Trends Genet. 1990; 6: 115–120. 2132730 10.1016/0168-9525(90)90125-p

[9] Palmer JD. Plastid chromosomes. structure and evolution. In cell culture and somatic cell genetics in plants Vol. 7A, The molecular biology of plastids, Vasil I.K., and Bograd L., eds. (Sandiego: Academic Press); 1991 pp: 5–53.

[10] Wicke S, Schneeweiss GM, dePamphilis CW, Müller KF, Quandt D. The evolution of the plastid chromosome in land plants: gene content, gene order, gene function. Plant Mol Biol. 2011; 76: 273–297. 10.1007/s11103-011-9762-4 21424877 PMC3104136

[11] Jansen RK, Palmer JD. A chloroplast DNA inversion markers and ancient evolutionary split in the sunflower family (Asteraceae). Proc Natl Acad Sci USA. 1987; 84: 5818–5822. 16593871 10.1073/pnas.84.16.5818 PMC298954

[12] Chumley TW, Palmer JD, Mower JP, Fourcade HM, Calie PJ, Boore JL et al The chloroplast genome sequence of *Pelargonium* Ⅹ *hortorum*: organization and evolution of the largest and most highly rearranged chloroplast genome of land plants. Mol Biol Evol. 2006; 23(11): 2175–2190. 16916942 10.1093/molbev/msl089

[13] Raubeson LA, Peery R, Chumley TW, Dzibek C, Fourcade HM, Boore JL et al Comparative chloroplast genomics: analyses including new sequences from the angiosperms *Nuphar advena* and *Ranunculus macranthus* . BMC Genomics. 2007; 8: 174 17573971 10.1186/1471-2164-8-174 PMC1925096

[14] Ma J, Yang B, Zhu W, Sun L, Tian J, Wang X. The complete chloroplast genome sequence of *Mahonia bealei* (Berberidaceae) reveals a significant expansion of the inverted repeat and phylogenetic relationship with other angiosperms. Gene. 2013; 528: 120–131. 10.1016/j.gene.2013.07.037 23900198

[15] Wu CS, Wang YN, Liu SM. Chloroplast genome (cpDNA) of *Cycas taitungensis* and 56 cp protein-coding genes of *Gnetum parvifolium*: insights in to cpDNA evolution and phylogeny of extant seed plants. Mol Biol Evol. 2007; 24:1366–1379. 17383970 10.1093/molbev/msm059

[16] Hirao T, Watanabe A, Kurita M, Kondo T, Takata K. Complete nucleotide sequence of the *Cryptomeria japonica* D. Don. chloroplast genome and comparative chloroplast genomics diversified genomic structure of coniferous species. BMC Plant Biol. 2008; 8: 70 10.1186/1471-2229-8-70 18570682 PMC2443145

[17] Plunkett GM, Dawnie SR. Expansion and contraction of the chloroplast inverted repeat in Apiaceae subfamily Apioideae. Syst Bot. 2000; 25(4): 648–667.

[18] Goulding SE, Olmstead RG, Morden CW, Wolfe KH. Ebb and flow of the chloroplast inverted repeat. Mol Gen Genet. 1996; 252: 195–206. 8804393 10.1007/BF02173220

[19] Wang R-J, Cheng C-L, Chang C-C, Wu C-L, Su T-M, Chaw S-M. Dynamics and evolution of the inverted repeat-large single copy junctions in the chloroplast genomes of monocots. BMC Evol Biol. 2008; 8: 36 10.1186/1471-2148-8-36 18237435 PMC2275221

[20] Saski C, Lee L-B, Daniell H, Wood TC, Tomkins J, Kim H-G et al Complete chloroplast genome sequence of *Glycine max* and comparative analyses with other legume genomes. Plant Mol Biol. 2005; 59:309–322. 16247559 10.1007/s11103-005-8882-0

[21] Millen RS, Olmstead RG, Adams KL, Palmer JD, Lao NT, Heggie L et al Many parallel losses of *infA* from chloroplast DNA during angiosperm evolution with multiple independent transfers to the nucleus. Plant Cell. 2001; 13: 645–658. 11251102 10.1105/tpc.13.3.645 PMC135507

[22] Jasnen RK, Saski C, Hansen AK, Daniell H. Complete plastid genome sequences of three Rosids (*Castanea*, *Prunus*, *Theobroma*): evidence for at least two independent transfers of *rpl22* to the nucleus. Mol Biol Evol. 2011; 28(1): 835–847. 10.1093/molbev/msq261 20935065 PMC3108605

[23] Ibrahim RIH, Azuma J-I, Sakamoto M. Complete nucleotide sequence of the Cotton (*Gossypium barbadense* L.) chloroplast genome with a comparative analysis of sequences among 9 dicot plants. Genes Genet Syst. 2006; 81: 311–321. 17159292 10.1266/ggs.81.311

[24] Sato S, Makamura Y, Kaneko T, Asamizu E, Tabata S. Complete structure of the chloroplast genome of *Arabidopsis thaliana* . DNA Res. 1999; 6: 283–290. 10574454 10.1093/dnares/6.5.283

[25] Hupfer H, Swiatek M, Hornung S, Herrmann RG, Maier RM, Chiu W-L et al Complete nucleotide sequence of the *Oenothera elata* plastid chromosome, representing plastome I of the five distinguishable Euoenthera plastomes. Mol Gen Genet. 2006; 263: 581–585. 10.1007/pl00008686 10852478

[26] Jansen RK, Cai Z, Raubeson LA, Daniell H, dePamphilis CW, Leebens-Mack J et al Analysis of 81 genes from 64 plastid genomes resolves relationships in angiosperms and identifies genome-scale evolutionary patterns. PNAS. 2007; 104(49): 19369–19374. 18048330 10.1073/pnas.0709121104 PMC2148296

[27] Wyman SK, Jansen RK, Boore HL. Automatic annotation of organellar genomes with DOGMA. Bioinformatics. 2004; 20: 3252–3255. 15180927 10.1093/bioinformatics/bth352

[28] Schattner P, Brooks AN, Lowe TM. The tRNAscan-SE, snoscan and snoGPS web servers for the detection of tRNAs and snoRNAs. Nucleic Acids Res. 2005; 33(2): W686–689. 15980563 10.1093/nar/gki366 PMC1160127

[29] Lohse M, Drechsel O, Bock R. OrganellarGenomeDRAW (OGDRAW): a tool for the easy generation of high-quality custom graphical maps of plastid and mitochondrial genomes. Curr Genet. 2009; 25(11): 1451–1452. 10.1007/s00294-007-0161-y 17957369

[30] Biomatters: Geneious v.6.1.7 [http://www.geneious.com/]

[31] Katoh K, Misawa K, Kuma K, Miyata T. MAFFT: a novel method for rapid multiple sequence alignment based on fast Fourier transform. Nucleic Acids Res. 2002; 30: 3059–3066. 12136088 10.1093/nar/gkf436 PMC135756

[32] Stamatakis A, Hoover P, Rougemont J. A rapid bootstrap algorithm for the RAxML web-server. Syst Biol. 2008; 75: 758–771. 10.1080/10635150802429642 18853362

[33] Untergrasser A, Cutcutache I, Koressaar T, Ye J, Faircloth BC, Remm M et al Primer3-new capabilities and interfaces. Nucleic Acids Res. 2012; 40(15): e115 22730293 10.1093/nar/gks596 PMC3424584

[34] Allan GC, Flores-Vergara MA, Krasynanski S, Kumar S, Thompson WF. A modified protocol for rapid DNA isolation from plant tissues using cetyltrithylammounium bromide. Nature Protocols. 2006; 1: 2320–5. 17406474 10.1038/nprot.2006.384

[35] Soltis DE, Gitzendanner MA, Soltis PS. A 567-taxon data set for angiosperms: the challenges posed by bayesian analyses of large data sets. Int J Plan Sci. 2007; 168: 137–157.

[36] Soltis DE, Soltis PS, Morgan DR, Swensen SM, Mullin BC, Dowd JM et al Chloroplast gene sequence data suggest a single origin of the predisposition for symbiotic nitrogen fixation in angiosperms. Proc Natk Acad Sci USA. 1995; 92: 2647–2651. 10.1073/pnas.92.7.2647 PMC42275 7708699

[37] Endress PK, Friis EM. Rosids-Reproductive structures, fossil and extant, and their bearing on deep relationships: Introduction. Pl Syst Evol. 2006; 260: 83–85.

[38] Daniell H, Wurdack KJ, Kanagaraj A, Lee S-B, Saski C, Jansen RK. The complete nucleotide sequence of the cassava (*Manihot esculenta*) chloroplast genome and the evolution of atpF in Malpighiales: RNA editing and multiple losses of group II intron. Theor Appl Genet. 2008; 116: 723–737. 10.1007/s00122-007-0706-y 18214421 PMC2587239

[39] Jung S, Cho I, Sosinski B, Abbott A, Main D. Comparative genomic sequence analysis of strawberry and other rosids reveals significant microsyntency. BMC Research Notes. 2010; 3: 168 10.1186/1756-0500-3-168 20565715 PMC2893199

[40] Ravi V, Khurana JP, Tyagi AK, Khurana P. The chloroplast genome of mulberry: complete nucleotide sequence, gene organization and comparative analysis. Tree Genet Genomes. 2006; 3: 49–59.

[41] Su H-J, Hongenhout SA, Al-Sadi AM, Kuo C-H. Complete chloroplast genome sequence of Omani Lime (*Citrus aurantiifolia*) and comparative analysis within the rosids. PLoS ONE. 2014; 9: 11. 10.1371/journal.pone.0113049 PMC4232571 25398081

[42] Wakasugi T, Tsudzuki J, Ito S, Nakashima K, Tsudzuki T, Sugiura M. Loss of all *ndh* genes as determined by sequencing the entire chloroplast genome of the black pine *Pinus thumbergii* . Proc Natl Acad Sci USA. 1994; 91: 9794–9798. 7937893 10.1073/pnas.91.21.9794 PMC44903

[43] Blazier JC, Guisinger MM, Jansen RK. Recent loss of plastid-encoded *ndh* genes within *Erodium* (Geraniaceae). Plant Mol Biol. 2011; 76: 263–272. 10.1007/s11103-011-9753-5 21327834

[44] Wicke S, Müller KF, de Pamphilis CW, Quandt D, Wuckett NJ, Zhang Y, Renner SS et al Mechanisms of functional and physical genome reduction in photosynthetic and nonphotosynthetuc parasitic plants of the broomrape family. Plant Cell. 2013, 25: 3711–3725. 10.1105/tpc.113.113373 24143802 PMC3877813

[45] Hansen DR, Dastidar SG, Penaflor C, Kuehl JV, Boore JL, Janse RK. Phylogenetic and evolutionary implications of complete chloroplast genome sequences for four early-diverging angiosperms: *Buxus* (Buxaceae), *Chloranthus* (Chloranthaceae), *Disocorea* (Dioscoreaceae), and *Illium* (Schisandraceae). Mol Phylogen Evol. 2007; 45: 547–563. 10.1016/j.ympev.2007.06.004 17644003

[46] Qian J, Song J, Gao H, Zhu Y, Xu J, Pang X et al The complete chloroplast genome sequence of the medicinal plant *Salvia miltiorrhiza* . PLoS ONE. 2013; 8(2): e57607 10.1371/journal.pone.0057607 23460883 PMC3584094

