# Supplementary material for: The Chloroplast Genome of Elaeagnus macrophylla and trnH Duplication Event in Elaeagnaceae
Source: PLoS One. 2015 Sep 22;10(9):e0138727. doi: 10.1371/journal.pone.0138727 (PMC4579063; doi:10.1371/journal.pone.0138727)
Supplement: S1 Table — (DOCX) [file pone.0138727.s001.docx]

Supplementary Material 1. Phylogenetic study taxa and Genbank accession number of references.

| Taxon | Accession number | Taxon | Accession number |
| --- | --- | --- | --- |
| *Nymphaea mexicana* | KF753633 | *Origanum vulgare* | JX880022 |
| *Buxus microphylla* | NC_016468 | *Salvia miltiorrhiza* | NC_020431 |
| *Vitis vinifera* | NC_007957 | *Prunus kensensis* | NC_023956 |
| *Spinacia oleracea* | NC_002202 | *Morus indica* | NC_008359 |
| *Eleutherococcus senticosus* | NC_016430 | *Elaeagnus macrophylla* | KF211788 |
| *Eucalyptus erythrocorys* | NC_022406 | *Lotus japonicas* | NC_002694 |
| *Nicotiana tabacum* | NC_01879 | *Theobroma cacao* | NC_014676 |
| *Datura stramonium* | NC_018117 | *Manihot esculenta* | EU117376 |
| *Atropa belladonna* | AJ316582 | *Populus euphratica* | NC_024747 |
| *Olea europaea* | NC_013707 | *Panax ginseng* | KF_431956 |
| *Boea hygrometrica* | NC_016468 | *Citrus sinensis* | NC_008334 |
| *Veronica insularis* | Preparing for submission | *Arabidopsis thaliana* | NC_000932 |
| *Scrophularia takesimensis* | NC_026202 | *Castanea mollissima* | NC_014674 |
| *Sesamum indicum* | JN637766 | *Gossypium barbadense* | NC_008641 |
