# Supplementary material for: The Chloroplast Genome of Elaeagnus macrophylla and trnH Duplication Event in Elaeagnaceae
Source: PLoS One. 2015 Sep 22;10(9):e0138727. doi: 10.1371/journal.pone.0138727 (PMC4579063; doi:10.1371/journal.pone.0138727)
Supplement: S2 Table — (DOCX) [file pone.0138727.s002.docx]

Supplementary Material 2. IR junction analysis taxa and accession number

| Taxon | Voucher | Accession number | |
| --- | --- | --- | --- |
|  |  | *rps19*- *rpl2* | *rpl2*-*psbA* |
| *Elaeagnus macrophylla* | YNUH EMC001 (YNUH) | KT167364 | KT167378 |
|  | 2101024c (YNUH) | KT167365 | KT167379 |
|  | 2091024a (YNUH) | KT167366 | KT167380 |
| *E. umbellata* | 2091010 (YNUH) | KT167367 | KT167381 |
| *E. maritima* | 2100220a (YNUH) | KT167368 | KT167382 |
| *E. glabra* | 2100221a (YNUH) | KT167369 | KT167383 |
| *E. pungens* | 2101018a (YNUH) | KT167390 | KT167384 |
| *Shephedia argentea* | Marian MacLord 678 (TEX) | KT167371 | KT167385 |
| *Rhamnus davurica* | RD1 (YNUH) | KT167372 | KT167386 |
| *R. ussurensis* | RU1 (YNUH) | KT167373 | KT167387 |
| *R. yoshinoi* | RY2 (YNUH) | KT167374 | KT167388 |
| *R. paravifolia* | RP1 (YNUH) | KT167375 | KT167389 |
| *R. koraiensis* | RK1 (YNUH) | KT167376 | KT167390 |
| *R. taqutii* | RT1 (YNUH) | KT167377 | KT167391 |

YNUH: herbarium of Yeungnam University (Korea)

TEX: Herbarium of Texas University (USA)
